# Intragraft FOXP3+ Cells and Continuous Banff Indices in T Cell-Mediated Kidney Allograft Rejection

**DOI:** 10.3390/medicina62091746

**Published:** 2026-09-10

**Authors:** Petar Đolonga, Petar Šenjug, Anja Stadnik, Ana Dunatov Huljev, Lada Zibar, Sandra Zekić Tomaš, Danica Galešić Ljubanović, Merica Glavina Durdov

**Affiliations:** 1 Department of Pathology, Judicial Medicine, and Cytology, University Hospital of Split, Spinčićeva 1, 21000 Split, Croatia; petar.dolonga@mefst.hr (P.Đ.); ana.dunatov@mefst.hr (A.D.H.); sandra.zekic-tomas@mefst.hr (S.Z.T.); 2 Department of Pathology, University of Split School of Medicine, Šoltanska 2, 21000 Split, Croatia; 3 Department of Nephropathology and Electron Microscopy, Dubrava University Hospital, Avenija Gojka Šuska 6, 10000 Zagreb, Croatia; petar.senjug@gmail.com (P.Š.); stadnik.anja@gmail.com (A.S.); danica.ljubanovic@mef.hr (D.G.L.); 4 Institute of Pathology, School of Medicine, University of Zagreb, Šalata 10, 10000 Zagreb, Croatia; 5 Department of Nephrology, Clinical Hospital Merkur, Zajčeva 19, 10000 Zagreb, Croatia; ladazibar@gmail.com; 6 Department of Pathophysiology, Faculty of Medicine, Josip Juraj Strossmayer University of Osijek, Huttlerova 4, 31000 Osijek, Croatia; 7 Faculty of Farmacy and Biochemistry, University of Zagreb, Ante Kovačića 1, 10000 Zagreb, Croatia

**Keywords:** T cell-mediated rejection, kidney allograft, Banff classification, FOXP3

## Abstract

*Background and Objectives*: FOXP3+ (Forkhead box P3) regulatory T cells contribute to immune tolerance after kidney transplantation, but their role in T cell-mediated rejection (TCMR) remains controversial. This study investigated the association between intragraft FOXP3+ cells and the recently introduced Banff-derived activity (AI) and chronicity index (CI) in biopsy-proven TCMR. *Materials and Methods*: This retrospective cross-sectional study included 119 kidney allograft biopsies diagnosed as acute or chronic active TCMR according to the Banff classification. FOXP3+ cells were assessed by immunohistochemistry and expressed as cortical cell density (cells/mm^2^). Associations between FOXP3+ cell density, Banff-derived indices, inflammatory cell densities, and clinical parameters were analyzed using non-parametric statistical methods. *Results*: FOXP3+ cells were detected in 17.6% of biopsies. FOXP3+ cell density was univariately associated with higher CI values (*ρ* = 0.228, *p* = 0.013) and chronic lesions interstitial fibrosis (*ci*) (*ρ* = 0.284, *p* = 0.002) and tubular atrophy (*ct*) (*ρ* = 0.246, *p* = 0.008). No association was observed with AI or activity lesions, except for a weak negative correlation with interstitial inflammation (*i*) (*ρ* = −0.209, *p* = 0.025). In multivariable analysis, FOXP3 positivity was not significantly associated with CI after adjustment for transplantation–biopsy interval (B = 0.568, 95% CI −0.741–1.876, *p* = 0.391). FOXP3+ cell density positively correlated with cluster of diferentiation (CD)4+ (*ρ* = 0.350, *p* < 0.001), CD8+ (*ρ* = 0.229, *p* = 0.018), and CD163+ (*ρ* = 0.207, *p* = 0.047) cell densities. No association with short-term graft outcome was observed. *Conclusions*: FOXP3+ cell infiltration was associated with chronic histological changes in unadjusted analyses, but this association did not remain statistically significant after adjustment for transplantation-to-biopsy interval. These preliminary findings suggest that FOXP3+ cell accumulation and chronic histological changes may represent parallel time-dependent phenomena and require confirmation in larger cohorts.

## 1. Introduction

T cell-mediated kidney transplant rejection (TCMR) is characterized by T cell-dominant inflammatory infiltrate that induces parenchymal injury manifested as tubulointerstitial and/or vascular inflammation [1]. According to the DeKAF study, acute TCMR (ATCMR) most frequently occurs within the first 12 months after transplantation, although it may also develop several years later [2]. The diagnosis of ATCMR is established by assessing specific histopathological lesions defined by the Banff classification for renal transplant pathology, including non-atrophic interstitial inflammation, tubulitis, and intimal arteritis. Chronic active TCMR (ChATCMR) is diagnosed when inflammation involves areas of interstitial fibrosis and tubular atrophy (i-IFTA ≥ 2) and is accompanied by sufficient total cortical inflammation (ti ≥ 2) and moderate (grade IA) or severe (grade IB) tubulitis. ChATCMR grade II is characterized by chronic allograft arteriopathy [3].

Increasing evidence suggests that kidney transplant rejection should be regarded as a continuum of immune-mediated injury arising from a complex interplay of alloimmune mechanisms, ranging from the well-established influence of donor–recipient human leukocyte antigen (HLA) mismatch to the increasingly recognized contribution of innate immune responses, rather than as discrete diagnostic categories [4]. This conceptual shift led to the establishment of the Banff Activity and Chronicity Indices Working Group, which aims to introduce continuous indices derived from active and chronic Banff lesion scores into diagnostic workflows, to better reflect the overlapping pathobiology of rejection phenotypes [5]. Recently, the activity index (AI) and chronicity index (CI) have been proposed as quantitative measures of acute and chronic histopathological injury, independent of rejection aetiology, with the aim of improving risk stratification in kidney transplant biopsies [6,7,8]. By integrating individual Banff lesions into cumulative measures of active and chronic histological injury, these indices provide a framework for examining whether intragraft immune-cell populations, including FOXP3+ cells, are associated with these components of the rejection process.

The inflammatory infiltrate in TCMR mainly consists of effector T-cells, but varying densities of other mononuclear and even polymorphonuclear leukocytes can be identified [9]. Particular attention is directed toward regulatory FOXP3 (forkhead box P3)-positive T lymphocytes, which exert immunoregulatory and anti-inflammatory effects through the suppression of effector immune responses [10]. Regulatory T cells (Tregs) are a subset of predominantly CD4+ lymphocytes that can be derived from thymocytes during the T cell selection process or induced in the periphery. In both cases, Tregs mediate immune tolerance [11]. In specific murine donor–recipient strain combinations, fully major histocompatibility complex (MHC)-mismatched kidney allografts can be spontaneously accepted without immunosuppression. In the DBA/2-to-B6 model, this early graft acceptance is dependent on FOXP3+ cells and is associated with the presence of Treg-rich organized lymphoid structures within the graft [12]. In contrast, evidence for a similar role of Tregs in human allografts remains limited and inconclusive. Some studies suggest that Tregs may serve as positive predictors of allograft function in patients receiving immunosuppressive therapy [13,14], whereas others report no significant differences in FOXP3+ cell infiltration between rejecting and non-rejecting allografts [15]. Whether intragraft FOXP3+ cell infiltration reflects the recently introduced continuous measures of histological activity and chronicity remains unknown.

The aim of this study was to investigate the association between intragraft FOXP3+ cell density and the recently introduced Banff-derived activity index (AI), chronicity index (CI), and their individual component lesions in biopsy-proven TCMR.

## 2. Materials and Methods

### 2.1. Study Cohort and Data Collection

The study analyzed 119 kidney transplant biopsies performed at the Department of Nephrology, Clinical Hospital Merkur, Zagreb, Croatia, between 2010 and 2025, and reviewed at the Department of Nephropathology and Electron Microscopy, Clinical Hospital Dubrava, Zagreb, Croatia. All histopathological lesions were independently evaluated and scored according to the Banff classification by two experienced renal pathologists. Discrepant scores were subsequently reviewed and resolved by consensus. For patients with multiple biopsies, only the first biopsy fulfilling the Banff diagnostic criteria for TCMR [3] was included in the primary study cohort. According to the Banff Classification for Renal Transplant Pathology, all included biopsies were classified as Banff category 4 (ATCMR or ChATCMR), whereas biopsies assigned to other Banff categories were excluded. Biopsies with insufficient residual tissue in the paraffin block for immunohistochemical analysis were also excluded. In a separate paired analysis, patients with an initial ATCMR biopsy who subsequently underwent biopsy demonstrating ChATCMR were identified. For these patients, the first ATCMR biopsy from the primary cohort and the first subsequent ChATCMR biopsy were analyzed as paired samples to evaluate temporal changes in FOXP3+ cell density. For patients with paired ATCMR and subsequent ChATCMR biopsies, the interval between the two biopsies was calculated for each patient.

Demographic and clinical characteristics, including the transplantation-to-biopsy interval, estimated glomerular filtration rate (eGFR) at the time of biopsy, and 1-year post-biopsy eGFR, were retrieved from histopathology reports and the electronic medical records of the Department of Nephrology, Clinical Hospital Merkur, Zagreb, Croatia. One-year post-biopsy eGFR data were available for 103 of 119 patients (86.6%), and these patients were included in the ΔeGFR analysis. No graft losses or deaths occurred during the first year after biopsy. Missing 1-year follow-up eGFR values were not imputed, and analyses involving 1-year eGFR and ΔeGFR were performed using available cases.

### 2.2. Continuous Activity and Chronicity Indices Calculation

AI and CI were calculated according to the mathematical models validated by Vaulet et al. [8] and Haas et al. [16]. AI was calculated using the following formula:

AI = *t* + *i* + *v* + *g* + *ptc* + 2 × *C4d*,

where *t* denotes tubulitis, *i* interstitial inflammation, *v* intimal arteritis, *g* glomerulitis, *ptc* peritubular capillaritis, and *C4d* (assessed by immunofluorescence) was coded as a binary variable (0 = negative, 1 = positive). CI was calculated as:

CI = *ci* + *ct* + *cv* + 2 × *cg*,

where *ci* denotes interstitial fibrosis, *ct* tubular atrophy, *cv* vascular fibrous intimal thickening, and *cg* transplant glomerulopathy (glomerular basement membrane double contours). All individual Banff lesion scores except C4d ranged from 0 to 3. AI and CI were analyzed as continuous variables, ranging from 0–17 to 0–15, respectively.

### 2.3. Immunohistochemical Staining and Quantification of Immune Cells

**Figure 1:**
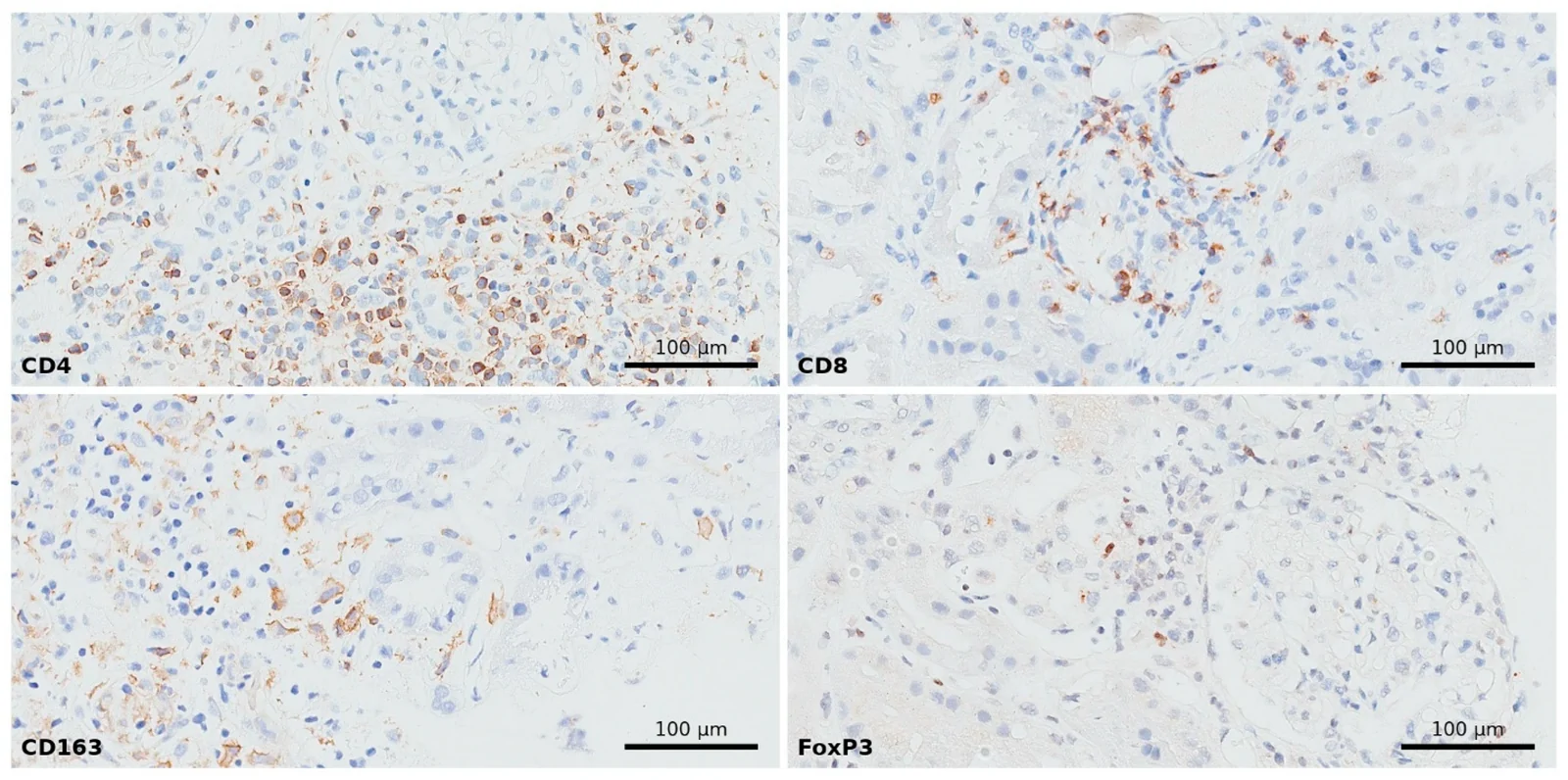
Representative immunohistochemical staining of CD4, CD8, C163, and FOXP3 in TCMR biopsies (×40 magnification). Positive staining is indicated by brown membranous signal for CD4, CD8, and CD163, and brown nuclear signal for FOXP3.

Paraffin-embedded tissue blocks were retrieved from the archive of the Department of Nephropathology and Electron Microscopy, Clinical Hospital Dubrava, Zagreb, Croatia. Sections were cut at 3 µm thickness and treated with the following antibodies: anti-CD4 (clone SP35, Ventana Roche, Tucson, AZ, USA), anti-CD8 (clone SP57, Ventana Roche, Tucson, AZ, USA), anti-CD163 (clone MRQ-26, Ventana Roche, Tucson, AZ, USA), and anti-FOXP3 (clone 236A/E7, Santa Cruz Biotechnology, Dallas, TX, USA). Detection was performed using the UltraView DAB Detection Kit (Ventana Roche, Tucson, AZ, USA). Positive staining was defined as brown membranous staining for CD4, CD8, and CD163, and brown nuclear staining for FOXP3 (Figure 1).

Slides were examined using an Olympus BX46 microscope (Olympus, Tokyo, Japan). Cortical surface area (mm^2^) was measured on each slide using the CellSens image analysis software version 3.1.1 (Olympus, Tokyo, Japan). Subsequently, CD4+, CD8+, and CD163+ cells were manually counted, and their cortical densities were expressed as the number of positive cells per mm^2^ of cortex. Given the generally weak and sparse nuclear staining of FOXP3+ cells, FOXP3 expression was initially assessed categorically. Cases with at least one FOXP3+ cell identified in a minimum of two non-overlapping ×40 high-power fields were classified as FOXP3-positive; all remaining cases were classified as FOXP3-negative. In FOXP3-positive cases, FOXP3+ cells were subsequently counted manually, and cortical density was expressed as the number of FOXP3+ cells per mm^2^ of cortex.

### 2.4. Statistical Analysis

Categorical variables were presented as absolute numbers (*n*) and percentages (%), and differences between groups were assessed using the chi-square (χ^2^) test. The distribution of continuous variables was assessed using the Shapiro–Wilk test. As most continuous variables were not normally distributed, they were presented as medians with interquartile ranges (IQRs), and differences between groups were evaluated using the Mann–Whitney U test. Correlations between continuous variables were assessed using Spearman’s rank correlation coefficient (*ρ*). A multivariable linear regression analysis was performed with the CI as the dependent variable and FOXP3 status and transplantation-to-biopsy interval as independent variables, to evaluate the association of each variable with CI while accounting for the other. Differences in FOXP3+ cell density between paired biopsies were evaluated using the Wilcoxon signed-rank test. A two-sided *p* value < 0.05 was considered statistically significant. All statistical analyses were performed using IBM SPSS Statistics for Windows, version 26 (IBM Corp., Armonk, NY, USA).

## 3. Results

### 3.1. Study Cohort Characteristics

This retrospective cross-sectional study included 119 indication kidney allograft biopsies diagnosed with TCMR, of which 85 (71.4%) were classified as ATCMR and 34 (28.6%) as ChATCMR. Baseline clinicopathological characteristics of the study cohort are presented in Table 1.

There were no significant differences in age or sex distribution between the ATCMR and ChATCMR groups. The median transplantation-to-biopsy interval was significantly longer in the ChATCMR group than in the ATCMR group (14.5 [IQR, 6–73] vs. 2 [IQR, 0.3–6] months, *p* < 0.001). Kidney function at biopsy also differed between groups (*p* = 0.015), with a lower median eGFR in the ATCMR group than in the ChATCMR group (35 [IQR, 16–59] vs. 49 [IQR, 34–60] mL/min/1.73 m^2^). The ΔeGFR over the first year after biopsy was significantly greater in the ATCMR group than in the ChATCMR group (12 [IQR, –1.8 to 33.8] vs. 0 [IQR, –10 to 8] mL/min/1.73 m^2^, *p* = 0.002), whereas eGFR at 1 year after biopsy did not differ significantly between the groups.

Both AI and CI differed significantly between the two TCMR phenotypes (*p* < 0.001 for both). AI was higher in the ATCMR group (median 5 [IQR, 5–7]) than in the ChATCMR group (median 3.5 [IQR, 3–6]), whereas CI was higher in the ChATCMR group (median 5 [IQR, 3–7.3]) than in the ATCMR group (median 2 [IQR, 0–2.8]). Individual Banff lesion scores are presented in Supplementary Table S1. Compared with ATCMR, ChATCMR biopsies showed significantly higher chronic lesion scores (*ci*, *ct*, *cv*, *ah*, *i-IFTA*), whereas ATCMR biopsies exhibited higher interstitial inflammation and tubulitis.

No significant differences were observed in the densities of CD4+, CD8+, CD163+, or FOXP3+ cells between the ATCMR and ChATCMR groups. However, after biopsies were categorized according to the presence or absence of FOXP3+ cells, a significantly higher proportion of ChATCMR biopsies were FOXP3-positive than ATCMR biopsies (29.4% vs. 12.9%, *p* = 0.033).

### 3.2. Clinicopathological Characteristics and Immune Cell Densities in Relation to FOXP3 Positivity

Because FOXP3 immunostaining yielded sparse and low-intensity nuclear staining, biopsies were initially categorized as FOXP3-positive and FOXP3-negative (Table 2). The transplantation-to-biopsy interval was significantly longer in the FOXP3-positive group than in the FOXP3-negative group (median 6 [IQR, 2.5–62] vs. 3 [IQR, 0.5–13.5] months, *p* = 0.011).

CI was significantly higher in FOXP3-positive biopsies than in FOXP3-negative biopsies (median 3 [IQR, 2.5–4.5] vs. 2 [IQR, 0–5], *p* = 0.004). eGFR at biopsy was also higher in FOXP3-positive biopsies (median 58 [IQR, 29–74] vs. 37 [IQR, 17.5–55.5] mL/min/1.73 m^2^, *p* = 0.048), whereas no significant differences were observed in eGFR at 1 year after biopsy or ΔeGFR during the first year after biopsy.

FOXP3-positive biopsies exhibited significantly higher CD4+ and CD8+ cell densities than FOXP3-negative biopsies (median 59.9 [IQR, 38.3–134.5] vs. 22.9 [IQR, 7–43], *p* = 0.001; and median 58 [IQR, 34.8–95.7] vs. 33.9 [IQR, 18.4–58.8], *p* = 0.034, respectively). Although CD163+ cell density tended to be higher in FOXP3-positive biopsies, the difference did not reach statistical significance (median 2.8 [IQR, 1.1–6.6] vs. 1.0 [IQR, 0.1–3.7], *p* = 0.081).

### 3.3. Correlations of FOXP3+ Cell Density with Banff-Derived Activity and Chronicity Lesions

To evaluate the associations between FOXP3+ cell density and Banff-derived AI and CI, as well as their individual component lesions, Spearman correlation analysis was performed (Table 3). FOXP3+ cell density showed a modest positive correlation with CI (Spearman’s *ρ* = 0.228, *p* = 0.013), as well as with the Banff chronicity lesions *ci* (Spearman’s *ρ* = 0.284, *p* = 0.002) and ct (Spearman’s *ρ* = 0.246, *p* = 0.008). No significant correlation was observed with the *cv* lesion. Among the activity lesions, FOXP3+ cell density demonstrated only a weak negative correlation with interstitial inflammation (Spearman’s *ρ* = –0.209, *p* = 0.025), whereas no significant correlations were observed with the remaining activity lesions or AI.

In the multivariable linear regression model (Table 4), transplantation-to-biopsy interval was significantly associated with higher CI (B = 0.052, 95% CI 0.037–0.067, *p* < 0.001), whereas FOXP3 positivity was not significantly associated with CI (B = 0.568, 95% CI −0.741–1.876, *p* = 0.391) (Table 4). The overall model was statistically significant (F(2, 94) = 27.681, *p* < 0.001), explaining 37.1% of the variance in CI (R^2^ = 0.371; adjusted R^2^ = 0.357). A sensitivity analysis using FOXP3+ cell density instead of FOXP3 positivity yielded similar results (Supplementary Table S2).

Additional correlations with clinical parameters and immune cell densities are presented in Supplementary Table S3. FOXP3+ cell density showed a modest positive correlation with the transplantation-to-biopsy interval and eGFR at biopsy, whereas no significant correlations were observed with eGFR at 1 year after biopsy or ΔeGFR. FOXP3+ cell density also correlated positively with CD4+, CD8+, and CD163+ cell densities.

### 3.4. Paired Biopsy Analysis

**Figure 2:**
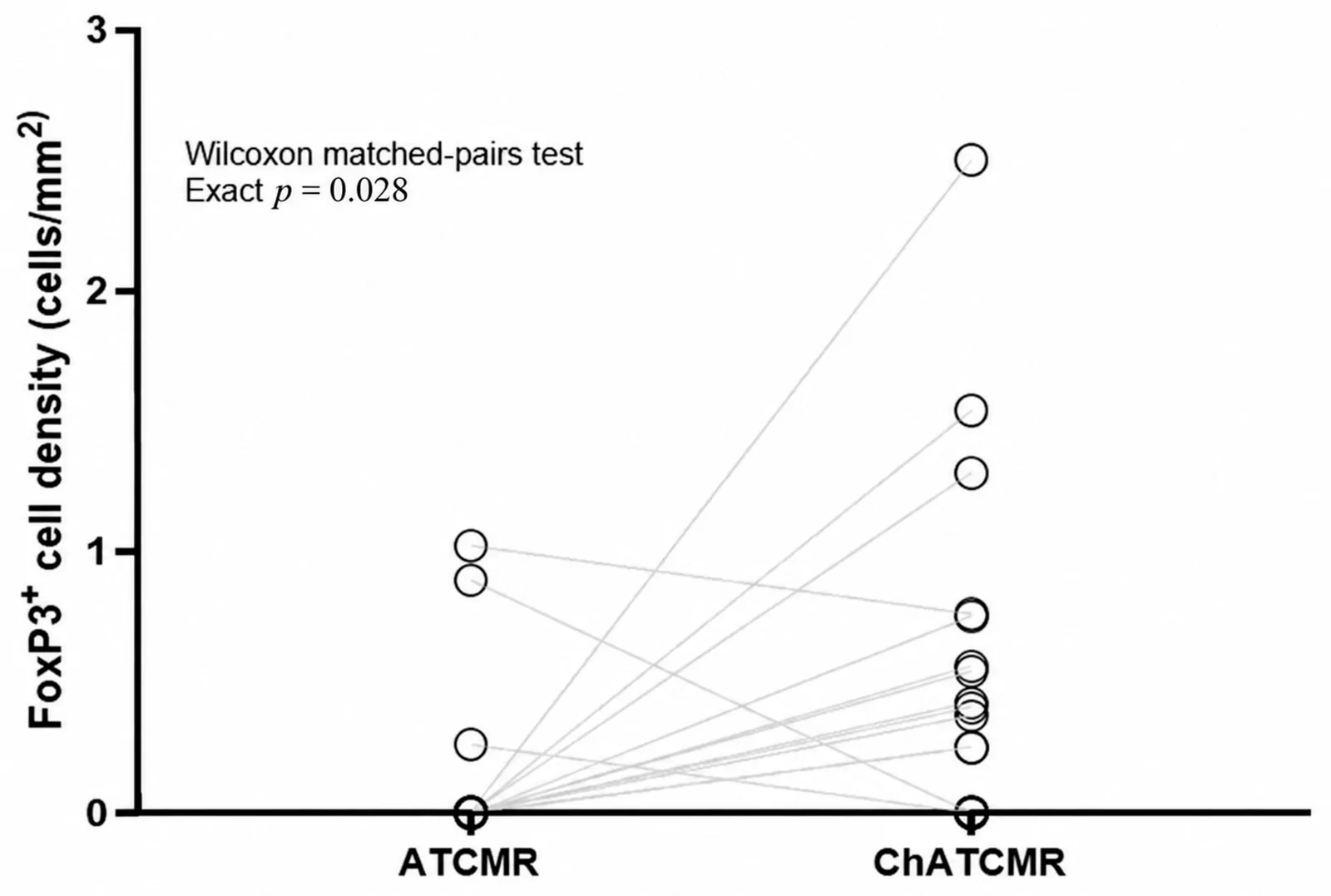
Changes in intragraft FOXP3+ cell density between paired acute (ATCMR) and chronic active (ChATCMR) T-cell mediated rejection biopsies (*n* = 30).

To evaluate changes in FOXP3+ cell density over time, paired biopsies from 30 patients initially diagnosed with ATCMR and subsequently with ChATCMR were compared using the Wilcoxon signed-rank test (Figure 2). The median interval between the initial ATCMR biopsy and subsequent ChATCMR biopsy was 11.6 months (IQR 6–58). FOXP3+ cell density was significantly higher in ChATCMR biopsies than in the corresponding ATCMR biopsies (median 0 [IQR, 0–0.5] vs. 0 [IQR, 0–0] cells/mm^2^, exact two-tailed *p* = 0.028). Despite the significant difference, most paired biopsies showed no detectable FOXP3+ cells. Among the 30 paired biopsies, FOXP3+ cell density increased in 11 (36.7%), decreased in 3 (10%), and remained unchanged in 16 (53.3%) cases.

## 4. Discussion

This retrospective cross-sectional study investigated the association between intragraft FOXP3+ cell density and acute and chronic histopathological lesions in kidney allograft biopsies with biopsy-proven TCMR. To our knowledge, this is the first study to evaluate FOXP3+ cell density in relation to the recently introduced Banff-derived AI and CI. Our findings showed that FOXP3+ cell density was associated with tubulointerstitial scarring in unadjusted analyses, reflected by higher CI scores and the individual Banff chronic lesions ci and ct. However, the association with CI did not remain statistically significant after adjustment for transplantation-to-biopsy interval. Furthermore, FOXP3+ cell density was not associated with short-term graft outcomes, apart from a weak positive correlation with eGFR at the time of biopsy.

We used immunohistochemistry to quantify FOXP3+ cell density in kidney allograft biopsies with TCMR. FOXP3+ cells were detected in only 17.6% of biopsies. Previous studies have reported highly variable mean cortical FOXP3+ cell densities [17,18,19]. Direct comparison with our findings is challenging, however, because previous studies have used partly different approaches to quantify and report FOXP3+ cell infiltration, and most have reported mean cell densities or numbers of positive cells per high-power field rather than the proportion of FOXP3-positive biopsies or median cell densities. This variability likely reflects methodological differences, including antibody selection, quantification approaches, study populations, and biopsy timing. In our cohort, the predominance of zero values resulted in a highly skewed distribution and a median FOXP3+ cell density of 0 cells/mm^2^. One possible explanation for the low frequency of FOXP3+ cells is the effect of immunosuppressive therapy. Previous studies have reported a trend toward lower intragraft FOXP3+ cell levels in patients receiving cyclosporine-based immunosuppression [17], while other immunosuppressive regimens have been associated with alterations in circulating follicular regulatory T-cell populations [20]. However, as individual post-transplant immunosuppressive regimens were not analyzed in the present study, this explanation remains speculative.

Another important factor that may have contributed to the low frequency of FOXP3+ cells is the timing of biopsy after transplantation. In our cohort, FOXP3-positive biopsies exhibited a significantly longer transplantation-to-biopsy interval than FOXP3-negative biopsies, while FOXP3+ cell density showed a weak-to-moderate positive correlation with transplantation-to-biopsy interval. Similarly, Bunnag et al. demonstrated that intragraft FOXP3 mRNA expression in kidney transplant biopsies was strongly influenced by the time elapsed after transplantation, suggesting that FOXP3+ cell infiltration is a time-dependent phenomenon that evolves alongside the inflammatory infiltrate [21]. Considering this temporal pattern together with the positive associations observed between FOXP3+ cell density and CD4+, CD8+, and CD163+ cell densities, our findings suggest that the presence of FOXP3+ cells in TCMR may reflect secondary accumulation within chronically inflamed allograft tissue rather than an isolated feature of rejection. This temporal hypothesis is further supported by our exploratory paired-biopsy analysis, in which ChATCMR biopsies tended to exhibit higher FOXP3+ cell density than the preceding ATCMR biopsies obtained from the same patients.

The main finding of this study was the modest univariate association of FOXP3+ cells, in both categorical and continuous analyses, with the Banff-derived CI and the individual chronic Banff lesion scores *ci* and *ct*. In contrast, FOXP3+ cell density was not associated with the Banff-derived AI, except for a weak negative correlation with the individual lesion score *i*. Bunnag et al. previously demonstrated that intragraft FOXP3+ cell density and FOXP3 mRNA expression were positively associated with the Banff lesion scores *i*, *t*, *ci*, and *ct* [21] whereas Yapici et al. reported associations with the lesion scores *t* and *ci*, but not with *i* or *ct* [22]. Importantly, these studies were conducted in heterogeneous biopsy cohorts that included antibody-mediated rejection, acute tubular injury, and calcineurin inhibitor toxicity, while only a limited proportion of biopsies represented TCMR. In contrast, our study exclusively evaluated biopsy-proven TCMR and, to our knowledge, is one of the first to investigate FOXP3+ cell density in relation to the recently introduced Banff-derived AI and CI.

The observed association between FOXP3+ cells and CI may reflect parallel time-associated phenomena, with both the development of chronic histological changes and the accumulation of FOXP3+ cells occurring with increasing time after transplantation, making it difficult to determine their relationship independently of time after transplantation. This interpretation is further supported by the multivariable analysis, in which the association between FOXP3 positivity and CI was no longer statistically significant after adjustment for transplantation-to-biopsy interval. Interestingly, similar observations have also been reported outside the transplant setting, where Allam et al. demonstrated a positive correlation between the proportion of FOXP3+ cells and the chronicity index in lupus nephritis [23]. Notably, FOXP3+ cell density was not associated with the overall AI, and among the individual Banff activity lesions, only a weak negative correlation with interstitial inflammation (*i*) was observed.

The prognostic significance of FOXP3 remains controversial. While several studies demonstrated that FOXP3 expression in peripheral blood, urine, or kidney allograft tissue was associated with graft survival, rejection reversibility, or progression of rejection and even poor outcomes [13,14,17,18,19,22,24,25], others failed to confirm these findings [21,26,27]. In our study, FOXP3+ cell density was weakly positively correlated with eGFR at the time of biopsy, while no significant associations were observed with short-term graft outcomes. The absence of an association with graft outcome in our cohort should be interpreted in the context of the relatively short follow-up period and the low frequency of detectable FOXP3+ cell infiltration, which may have limited our ability to identify prognostic associations. The prognostic relevance of intragraft immune-cell populations has also been examined using broader immunophenotypic approaches. Vaulet et al. evaluated multiple immune-cell populations and reported that conventional immune-cell populations generally lacked both discriminatory value between rejection phenotypes and independent prognostic significance, with CD8Temra (terminally differentiated effector memory cells re-expressing CD45RA) representing the only immune subset consistently associated with long-term graft outcomes [28].

Although this study included a relatively large cohort of biopsy-proven TCMR, several limitations should be acknowledged. First, the retrospective cross-sectional design precludes conclusions regarding causality between FOXP3+ cell infiltration and chronic histological injury. Second, FOXP3 immunohistochemistry yielded sparse positive cells in the majority of biopsies, resulting in a highly skewed distribution with a substantial number of tied zero values, which limits the interpretation of correlation analyses using continuous FOXP3+ cell density. In addition, identification of regulatory T cells was based solely on FOXP3 immunohistochemistry. Although FOXP3 is the most widely used tissue marker of regulatory T cells, additional phenotypic or molecular markers could further improve their characterization. Third, the relatively small ChATCMR subgroup and the differences in transplantation-to-biopsy intervals between the ATCMR and ChATCMR groups limit the interpretation of direct between-group comparisons. Consequently, these subgroup findings should be considered preliminary and hypothesis-generating. Fourth, detailed data regarding individual post-biopsy immunosuppressive treatment were not available, precluding assessment of its potential influence on intragraft FOXP3+ cell infiltration. Finally, given the apparent time-dependent nature of FOXP3+ cell infiltration, future multicenter studies including larger TCMR cohorts and complementary molecular approaches are warranted to further clarify the relationship between Banff-derived activity and chronicity indices and the intragraft immune microenvironment.

## 5. Conclusions

In unadjusted analyses, FOXP3+ cell infiltration was associated with the Banff-derived CI and the individual chronic Banff lesions *ci* and *ct*. However, the association between FOXP3 positivity and CI did not remain statistically significant after adjustment for the transplantation-to-biopsy interval, suggesting that FOXP3+ cell accumulation and chronic histological changes may represent parallel time-dependent phenomena that cannot be clearly distinguished in the present study. In contrast, no association was observed with the Banff-derived AI, while among the individual activity lesions, only a weak negative correlation with interstitial inflammation was found. Given the limitations of the present study, these findings should be considered preliminary and hypothesis-generating and require confirmation in larger independent cohorts.

## Figures and Tables

**Table 1:** Characteristics of the study cohort according to TCMR phenotype.

Variable		Total (*n* = 119)	ATCMR (*n* = 85, 71.4%)	ChATCMR (*n* = 34, 28.6%)	*p*
		*n* (%) or median (IQR)			
Sex	Male	84 (70.6)	63 (74.1)	21 (61.8)	0.182
	Female	35 (29.4)	22 (25.9)	13 (38.2)	
Age (years)		49 (40.5–60.3)	52 (43.3–60)	49 (38.8–56)	0.196
Transplantation-to-biopsy interval (months)		4.5 (0.5–12.3)	2 (0.3–6)	14.5 (6–73)	<0.001 *
eGFR at biopsy (mL/min/1.73 m^2^)		40.5 (23.5–59)	35 (16–59)	49 (34–60)	0.015 *
eGFR at 1-year post-biopsy (mL/min/1.73 m^2^)		51 (39–68)	53.5 (42.3–68.8)	47 (37–64)	0.189
ΔeGFR (mL/min/1.73 m^2^)		7 (−5–18)	12 (−1.8–33.8)	0 (−10–8)	0.002 *
Activity index		5 (4–7)	5 (5–7)	3.5 (3–6)	<0.001 *
Chronicity index		2 (0–4)	2 (0–2.8)	5 (3–7.3)	<0.001 *
CD4^+^ cells/mm^2^		27.4 (8–56.7)	30.8 (7–73.8)	24.7 (14.5–48.2)	0.992
CD8^+^ cells/mm^2^		38.6 (22.1–76.1)	37 (20–65.6)	34 (19.5–57.9)	0.778
CD163^+^ cells/mm^2^		1.3 (0.2–5.5)	1.3 (0.2–5.9)	1.1 (0.4–4.1)	0.523
FoxP3^+^ cells/mm^2^		0 (0–0)	0 (0–0)	0 (0–0.48)	0.062
FoxP3-positive biopsies		21 (17.6)	11 (12.9)	10 (29.4)	0.033 *

Categorical variables were compared using Pearson’s χ^2^ test, and continuous variables using the Mann–Whitney U test. IQR—interquartile range; ATCMR—acute T-cell-mediated rejection; ChATCMR—chronic active T-cell-mediated rejection; eGFR—estimated Glomerular Filtration Rate; FoxP3—forkhead box P3; ΔeGFR = eGFR 1-year post-biopsy-eGFR at biopsy; *—*p* < 0.05.

**Table 2:** Clinicopathological characteristics according to FOXP3 positivity.

Variable	FOXP3-Negative Biopsies (*n* = 98, 82.4%)	FOXP3-Positive Biopsies (*n* = 21, 17.6%)	*p*
	median (IQR)		
Transplantation-to-biopsy interval (months)	3 (0.5–13.5)	6 (2.5–62)	0.011 *
eGFR at biopsy (mL/min/1.73 m^2^)	37 (17.5–55.5)	58 (29–74)	0.048 *
eGFR at 1-year post-biopsy (mL/min/1.73 m^2^)	51 (40.5–67)	53 (40–76.5)	0.897
ΔeGFR (mL/min/1.73 m^2^)	7 (−6–31)	8 (0–14)	0.348
Activity index	5 (3–6)	5 (3–5.5)	0.241
Chronicity index	2 (0–5)	3 (2.5–4.5)	0.004 *
CD4^+^ cells/mm^2^	22.9 (7–43)	59.9 (38.3–134.5)	0.001 *
CD8^+^ cells/mm^2^	33.9 (18.4–58.8)	58 (34.8–95.7)	0.034 *
CD163^+^ cells/mm^2^	1 (0.1–3.7)	2.8 (1.1–6.6)	0.081

Continuous variables were compared using the Mann–Whitney U test. IQR—interquartile range; eGFR—estimated Glomerular Filtration Rate; FoxP3—forkhead box P3; ΔeGFR = eGFR 1-year post-biopsy–eGFR at biopsy; *—*p* < 0.05.

**Table 3:** Spearman correlations between FOXP3+ cell density and Banff-derived activity and chronicity indices and their individual Banff lesion scores.

Variable	FOXP3+ Cell Density (Cells/mm^2^)	
	Spearman *ρ*	*p*
Activity index	−0.097	0.300
Interstitial inflammation (*i*)	−0.209	0.025 *
Tubulitis (*t*)	0.029	0.755
Intimal arteritis (*v*)	−0.023	0.807
Glomerulitis (*g*)	−0.114	0.224
Peritubular capilaritis (*ptc*)	−0.136	0.145
Chronicity index	0.228	0.013 *
Interstitial fibrosis (*ci*)	0.284	0.002 *
Tubular atrophy (*ct*)	0.246	0.008 *
Vascular fibrous intimal thickening (*cv*)	0.081	0.391

C4d and cg are were 0 in all included biopsies; FoxP3—forkhead box P3; *—*p* < 0.05.

**Table 4:** Multivariable linear regression analysis of factors associated with the chronicity index.

Predictor	B	SE	95% CI	*p*
Transplantation-to-biopsy interval (months)	0.052	0.007	0.037–0.067	<0.001 *
FoxP3 positivity	0.568	0.659	−0.741–1.876	0.391

B—unstandardised regression coefficient; SE—standard error; CI—confidence interval; *—*p* < 0.05.

## Data Availability

The datasets generated and/or analyzed during the current study are available from the corresponding author on reasonable request.

## Supplementary Materials

The following supporting information can be downloaded at https://www.mdpi.com/article/10.3390/medicina62091746/s1, Table S1: Distribution of individual Banff lesion scores according to TCMR phenotype; Table S2: Multivariable linear regression analysis of FOXP3+ cell density and transplantation-to-biopsy interval in relation to the chronicity index. Table S3: Spearman correlations between FOXP3-positive cell density and clinical and immunohistochemical parameters.

## Abbreviations

The following abbreviations are used in this manuscript:

TCMR	T cell-mediated rejection
ATCMR	Acute T cell-mediated rejection
ChATCMR	Chronic active T cell-mediated rejection
HLA	Human leukocyte antigen
AI	Activity index
CI	Chronicity index
FOXP3	Forkhead box P3
eGFR	Estimated glomerular filtration rate
IQR	Interquartile range
