# Supplementary material for: Intragraft FOXP3+ Cells and Continuous Banff Indices in T Cell-Mediated Kidney Allograft Rejection
Source: Medicina (Kaunas). 2026 Sep 10;62(9):1746. doi: 10.3390/medicina62091746 (PMC13609503; doi:10.3390/medicina62091746)
Supplement: Supplementary file 1 [file medicina-62-01746-s001.zip › medicina-4460423-supplementary.pdf]

Supplementary Table S1. Distribution of individual Banff lesion scores according to TCMR phenotype.

VARIABLE	ATCMR	ChATCMR	*p*
	median (IQR)		
Interstitial inflammation (*i*)	2 (2 – 2)	1 (0 – 1)	< 0.001*
Tubulitis (*t*)	3 (2 –3)	2 (2 – 3)	0.035*
Intimal arteritis (*v*)	0 (0 – 0)	0 (0 – 0)	0.128
Glomerulitis (*g*)	0 (0 – 0)	0 (0 – 0)	0.755
Peritubular capilaritis (*ptc*)	0 (0 – 1)	0 (0 – 0)	0.248
Interstitial fibrosis (*ci*)	0 (0 – 1)	2 (1 – 2)	< 0.001*
Tubular atrophy (*ct*)	1 (0 – 1)	2 (1 – 2)	< 0.001*
Vascular fibrous intimal thickening (*cv*)	0 (0 – 1)	1 (0 – 3)	< 0.001*
Mesangial matrix expansion (*mm*)	0 (0 – 0)	0 (0 – 0)	0.010*
Arteriolar hyalionsis (*ah*)	1 (0 – 2)	3 (1 – 3)	< 0.001*
Total inflammation (*ti*)	2 (0 – 2)	2 (2 – 2.8)	0.044*
Inflammation in area of IFTA (*i-IFTA*)	0 (0 – 0)	3 (2 – 3)	< 0.001*

Ordinal variables were compared using the Mann–Whitney U test. IQR—interquartile range; ATCMR—acute T-cell mediated rejection; ChATCMR—chronic active T-cell mediated rejection; IFTA—interstitial fibrosis and tubular atrophy; *—$p < 0.05$.

Supplementary Table S2. Multivariable linear regression analysis of FOXP3+ cell density and transplantation-to-biopsy interval in relation to the chronicity index.

PREDICTOR	B	SE	95 % CI	p
Transplantation-to-biopsy interval (months)	0.053	0.007	0.039 – 0.068	<0.001*
FoxP3+ cells/mm^2	0.025	0.196	-0.364 – 0.413	0.871

B—unstandardised regression coefficient; SE—standard error; CI—confidence interval; *—p < 0.05.

Supplementary Table S3. Spearman correlations between FOXP3-positive cell density and clinical and immunohistochemical parameters.

VARIABLE	FOXP3+ cell density (cells/mm2)	
	Spearman ρ	p
Transplantation-to-biopsy interval (months)	0.233	0.021*
eGFR at biopsy (mL/min/1.73 m^2)	0.200	0.041*
eGFR at 1-year post-biopsy (mL/min/1.73 m^2)	0.010	0.920
ΔeGFR (mL/min/1.73 m^2)	-0.074	0.460
$CD4^+$ cells/mm^2	0.350	< 0.001*
$CD8^+$ cells/mm^2	0.229	0.018*
$CD163^+$ cells/mm^2	0.207	0.047*

eGFR—estimated Glomerular Filtration Rate; FoxP3—*forkhead box P3*; ΔeGFR = eGFR 1-year post-biopsy - eGFR at biopsy; *—p < 0.05.
